# Potent antitumor activity of oncolytic adenovirus expressing Beclin-1 via induction of autophagic cell death in leukemia

**DOI:** 10.18632/oncotarget.1018

**Published:** 2013-06-03

**Authors:** Yin Tong, Liangshun You, Hui Liu, Lu Li, Haitao Meng, Qijun Qian, Wenbin Qian

**Affiliations:** ^1^ Institute of Hematology, the First Affiliated Hospital, College of Medicine, Zhejiang University, P.R. China; ^2^ Laboratory of Viral and Gene Therapy, Eastern Hepatobiliary Surgery Hospital, Second Military Medical University, P.R. China

**Keywords:** leukemia, oncolytic adenovirus, autophagy, Beclin-1, autophagic cell death, UVRAG

## Abstract

An attractive strategy among adenovirus-based oncolytic systems is to design adenoviral vectors to express pro-apoptotic genes, in which this gene-virotherapy approach significantly enhances tumor cell death by activating apoptotic pathways. However, the existence of cancer cells with apoptotic defects is one of the major obstacles in gene-virotherapy. Here, we investigated whether a strategy that combines the oncolytic effects of an adenoviral vector with simultaneous expression of Beclin-1, an autophagy gene, offers a therapeutic advantage for leukemia. A Beclin-1 cDNA was cloned in an oncolytic adenovirus with chimeric Ad5/11 fiber (SG511-BECN). SG511-BECN treatment induced significant autophagic cell death, and resulted in enhanced cell killing in a variety of leukemic cell lines and primary leukemic blasts. SG511-BECN effects were seen in chronic myeloid leukemia and acute myeloid leukemia with resistance to imatinib or chemotherapy, but exhibited much less cytotoxicity on normal cells. The SG511-BECN-induced autophagic cell death could be partially reversed by RNA interference knockdown of UVRAG, ATG5, and ATG7. We also showed that SG511-BECN strongly inhibited the growth of leukemic progenitors in vitro. In murine leukemia models, SG511-BECN prolonged the survival and decreased the xenograft tumor size by inducing autophagic cell death. Our results suggest that infection of leukemia cells with an oncolytic adenovirus overexpressing Beclin-1 can induce significant autophagic cell death and provide a new strategy for the elimination of leukemic cells via a unique mechanism of action distinct from apoptosis.

## INTRODUCTION

Cancer virotherapy is a tumor-specific strategy, in which viruses are engineered to preferentially replicate in tumor cells and destroy it by lysis, either through targeted alterations in the cancer cells such as p53 mutation, viral deletion, tissue-specific transcriptional control or tumor-specific receptors [1-3]. Among available virotherapies, conditionally replicating adenoviruse (CRAd) is attractive because of its several attributes including lytic replication, efficient genome transfer, and excellent patient safety in clinical trials [4,5]. However, the previous clinical trials showed that *in vivo* efficacy of even CRAds is generally not sufficient for cancer therapy in clinic. Therefore, there are many attempts have been made to enhance the therapeutic index of CRAds. Two main strategies are currently being used to engineer CRAds to make them more selective and cytotoxic to tumor cells. The first approach is the creation of chimeric vectors, where the whole fiber or only the knob region is replaced with that of another serotype of adenovirus (Ad), which has led to decreased hepatotoxicity following virus administration attributed to less liver tropism, and increased infectivity of target tumor by coxsackie adenovirus receptor (CAR)-independent transduction [6-9]. The clinical trials of chimeric CRAd show evidence of antitumor activity ranging from 61% to 67% and viral replication in the blood when the patients with advanced cancers were treated intratumorally or intravenously with chimeric viruses [10,11]. In addition, chimeric CRAds might be effective against cancer-initiating cells or cancer stem cells (CSC) [6,12]. For example, Ad5/3-Delta24, a capsid-modified CRAd, has been demonstrated to effectively kill CD44^+^CD24^−/low^ breast CSCs *in vitro* and *in vivo* [13]. Previously, we reported that a fiber-modified CRAd (Ad5/35) could permit CAR-independent cell entry and induce selective cytopathic effects in human leukemic cells [8]. Taken together, these studies suggest the possibility of clinical application of virotherapy for leukemia.

The second strategy is based on the insertion of therapeutic genes into the genome of a modified CRAd, thereby creating a so-called gene-virotherapy. Gene-virotherapy shares the advantages of gene therapy and virotherapy, which can not only directly kill cancer cells by oncolysis, but also augment the copies of therapeutic genes by replication of the virus, resulting in longer transgene expression within tumors and potent activity against cancers [14-16]. Up to now, CRAds have been armed with a variety of transgenes that include tumor suppressor, pro-apoptotic, anti-angiogenic, immunomodulatory, and suicide genes [17,18]. We previously generated a series of E1B-55K deleted CRAds armed with different pro-apoptotic genes, such as tumor necrosis factor-related apoptosis-inducing ligand (TRAIL), p53, and interleukin-24, and demonstrated that the combination of pro-apoptotic or tumor suppressor genes and viral oncolysis yielded an additive cytotoxic effect on cancer cells. These viruses also proved more effective than the unarmed control vector at suppressing tumor growth *in vivo*, and prolonged survival in tumor or leukemia models [8,19,20]. However, cancer is a complex disorder associated with defects in multiple signaling pathways that confer resistance to apoptosis, suggesting the need for another innovative strategy [20,21]. Recent studies have demonstrated that autophagic cell death may serve as a novel way to eliminate tumor cells with defective apoptosis [22-26]. Therefore, we reasoned that arming CRAds with the genes inducing autophagic cell death could kill effectively cancer cells, especially cancer cells resistant to apoptosis, and represent an attractive prospect.

In this study, we combined the Beclin-1 gene therapy that induces autophagic cell death with SG511 vector (a new Ad5/11 fiber chimeric CRAd). The studies show that overexpression of Beclin-1 significantly enhanced the killing effect of the virus on leukemia cell lines and primary leukemic cells, in which cytotoxic activity of the parental virus without Beclin-1 gene was weak overall. In addition, Beclin-1 expression also strongly inhibited the growth of leukemic progenitors and prolonged the survival of leukemic xenograft-bearing animals by inducing autophagic cell death. From these findings, it is clear that the strategy of inducing autophagic cell death can be successfully linked with oncolytic viral approaches in the quest for an efficacious therapeutic approach for treating leukemias.

## RESULTS

### Construction and characterization of a chimeric, Beclin-1-armed CRAd

**Figure 1:**
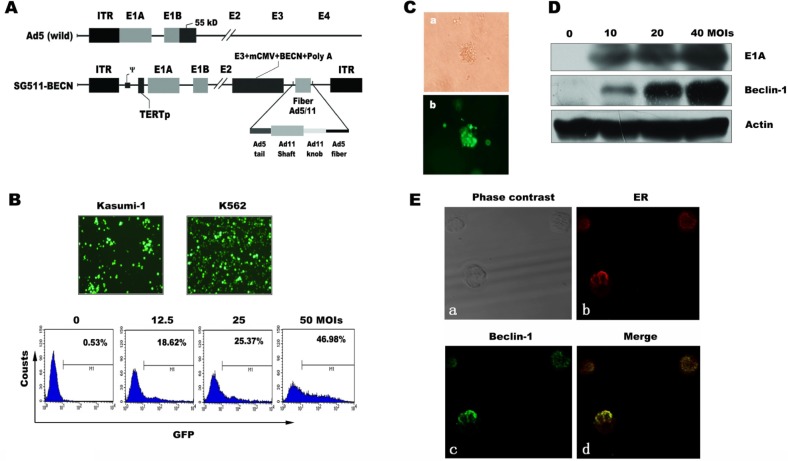
Schematic diagram of adenoviral constructs and CRAd infectivity in leukemic cells (A) Compared to wild-type Ad5, SG511-BECN contains a human telomerase reverse transcriptase (hTERT) promoter-controlled E1 expression cassette in forward orientation, an E1B 55-kDa deletion, and Beclin-1 transgene in E3 region. The fiber was modified by an Ad5/11 chimeric fiber. (B) Kusumi-1 and K562 cells were infected with 50 MOI of SG511-GFP for 24 h and then observed under a fluorescence microscope (200×) (top panel). K562 cells were treated with the indicated amount of the virus for 24 h and then collected for analysis by FACS (bottom panel). (C) Bone marrow cells obtained from a patient with AML were infected with SG511-GFP at an MOI of 50, and then cultured for 5 days in colony culture assay. Photographs were viewed under a light microscope and fluorescence microscope, respectively (400×). (D) Beclin-1 and E1A expression was determined by Western blotting of K562 cells infected with SG511-BECN at the indicated concentrations for 48 h. Actin was Western blotted for equal loading. (E) Following SG511-BECN treatment for 48 h, Beclin-1 (green) and ER (red) in K562 cells were detected by confocal immunofluorescence microscopy. Representative data of three experiments is shown.

Schematic structures of the genomes of SG511-BECN, along with wild Ad5, are shown in Figure 1A. The Beclin-1 transgene was engineered into the E3 region of the genome under the control of cytomegalovirus promoter. K562 and Kasumi-1 were used to evaluate the infectious efficiency of SG511 that is a similar virus without Beclin-1. CRAd containing the GFP gene (SG511-GFP)-infected cells was photographed using a fluorescence microscopy. As shown in Figure 1B, the leukemic cells were easily transduced by the virus. FACS analysis showed that the proportion of GFP-positive K562 cells increased in a dose-dependent manner after SG511-GFP infection. To determine the infection efficiency of SG511 on primary leukemic cells, the blasts from 7 AML patients were treated with SG511-GFP. The average portion of GFP-expressing cells was 68.77% at 2 days post-infection (MOI=50) (Supplementary Fig. 1A). Moreover, leukemic blasts from an AML patient were treated with SG511-GFP (MOI=50), and colony forming unit-leukemia (CFU-L) was assessed in methylcellulose culture. The cells within CFU-L showed bright green fluorescence dots (Fig. 1C), suggesting that this chimeric CRAd could effectively infect leukemic progenitor cells.

To investigate whether the infection of leukemia cells with SG511-BECN can produce Beclin-1 protein, K562 cells were exposed to the virus for 48 h and subjected to Western blot analysis. Results showed the dose-dependent increase of Beclin-1 and adenovirus E1A expression, respectively (Fig. 1D). To examine whether expression of Beclin-1 could hinder replication of CRAd, a progeny assay was performed in the supernatants of K562 cells infected with SG511 and SG511-BECN, respectively. Results demonstrated that the yield of virus production was equivalent in the two treatment groups (data not shown). Next we examined the subcellular distribution of Beclin-1 in the infected K562 cells using antibodies to costain Beclin-1 and Calreticulin. Confocal immunofluorescence analysis revealed the colocalization of Beclin-1 protein with Calreticulin, an endoplasmic reticulum (ER) protein (Fig. 1E), consistent with the results of previous studies [27,28].

### SG511-BECN induced autophagic cell death in leukemia cells

**Figure 2:**
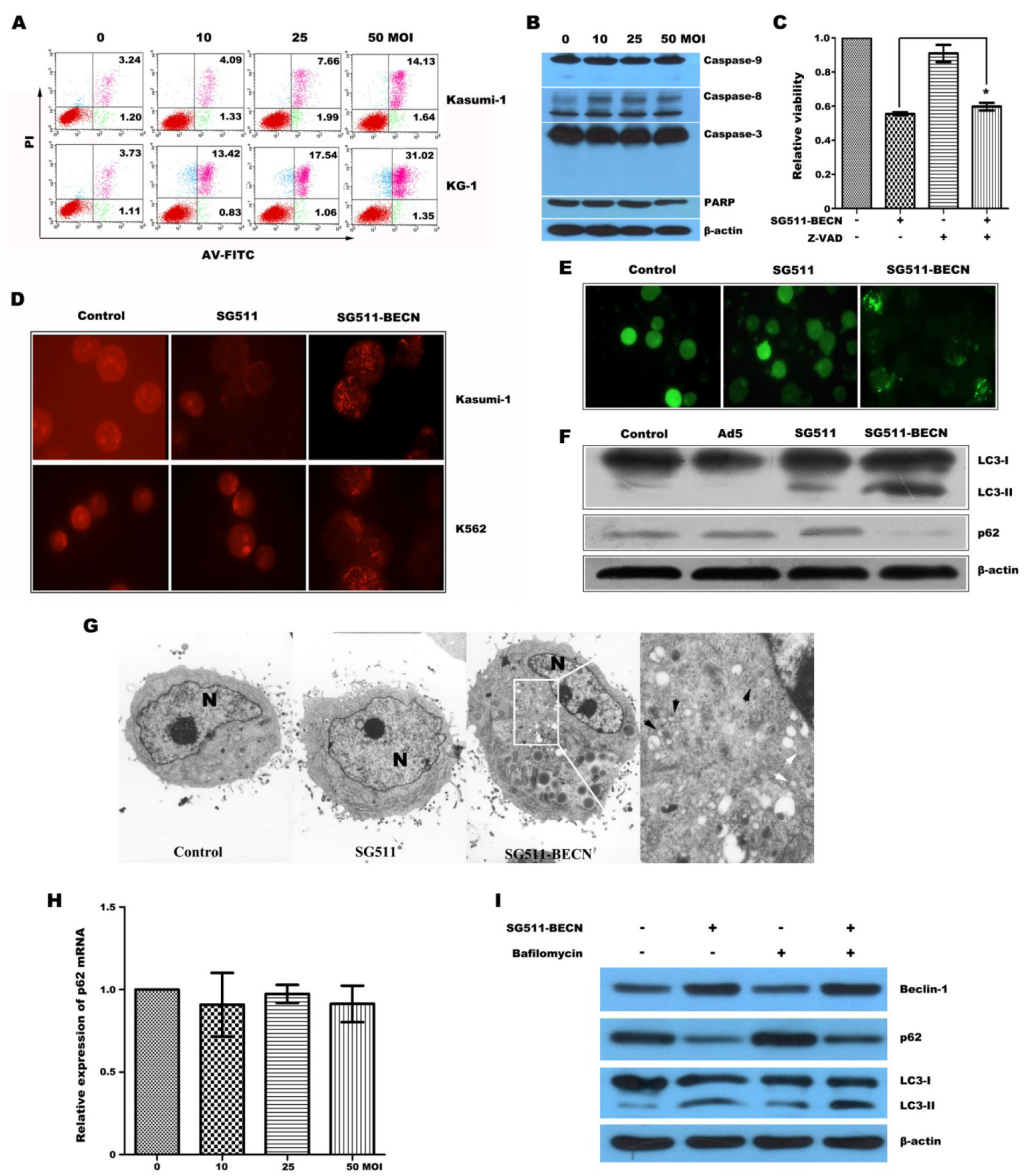
SG511-BECN virus induced cell death involves autophagy in leukemia cells (A) Kasumi-1 and KG-1 cells were treated with SG511-BECN at the doses indicated. Two days later, cell death was assessed by FACS analysis of annexin V- and PI staining. (B) K562 cells were treated with SG511-BECN at the indicated doses for 48 h before cell lysates were immunoblotted for the protein indicated. (C) K562 cells were incubated with SG511-BECN, or incubated with z-VAD (10 ìM) and SG511-BECN for 72 h. Then, cell viability was determined by an MTT assay. *represents *P*>0.05 (D) Formation of AVOs was determined after leukemic cells were infected with or without SG511, or SG511-BECN at an MOI of 50 for 48 h. (E) Formation of GFP-LC3 vacules (dots) was determined after K562 cells were stably transfected with GFP-LC3 vector and treated with 50 MOI of SG511 or SG511-BECN for 48 h. (F) K562 cells were infected with or without the indicted viruses (50 MOI) for 48 h. The cell lysates were harvested and analyzed by Western blotting using anti-LC3 and anti-p62 antibodies. (G) Kasumi-1 cells treated with SG511 and SG511-BECN, respectively and observed under TEM (×4200). There are two types of vacuole in the cytoplasm of SG511-BECN-treated cells: dense multilamellar bodies (white arrows) and inclusion bodies (black arrows). A representative of two separate experiments is shown. (H) qRT-PCR analysis monitoring p62 expression in K562 cells treated with SG511-BECN. Bars represent SD. (I) K562 cells were infected with 50 MOI SG511-BECN, alone or in combination with 20 nM BafA1. Total cell lysates were immunoblotted with anti-LC3, anti-p62, or anti-Actin antibodies, as indicated.

Based on the overexpression of Beclin-1 in SG511-BECN-infected cells, we sought to determine whether treatment of cells with the virus results in autophagic cell death. Figure 2A shows that SG511-BECN caused a dose-dependent non-apoptotic cell death that is characteristic of annexin V^+^ and PI^+^ [25]. Furthermore, significant activation of caspase pathway was not observed (Fig. 2B). Co-treatment the broad caspase inhibitor z-VAD-fmk did not block SG511-BECN-induced inhibition of cellular viability, suggesting that this virus activates caspase-independent cell death (Fig. 2C). Autophagy is characterized by the formation of AVOs and the location of microtubule-associated protein LC3 on autophagosomes [23]. Thus, we detected autophagy by measuring: (1) formation of AVOs by FACS; (2) electron microscopy of AVOs; (3) formation of GFP-LC3-labeled vacuoles by transfection and fluorescent microscopy; and (4) conversion of LC3-I to LC3-II by Western blot. As expected, SG511-BECN significantly induced AVO formation in Kasumi-1 and K562 cells over a 48-hour time course, whereas the SG511 vector did not (Fig. 2D). Furthermore, SG511-BECN also resulted in a significant formation of GFP-LC3-labled vacuoles (Fig. 2E). Strong up-regulation of LC3-II and degradation of p62 was observed upon SG511-BECN treatment, but not in PBS- or wild Ad5-treated cells; however a weak up-regulation of LC3-II was observed in the cells infected with SG511 (Fig. 2F). In agreement with those results, EM showed many autophagic vacuoles in the cells treated with SG511-BECN. In contrast, these vacuoles were not seen in the PBS- or SG511-treated cells (Fig. 2G). Quantitative real-time PCR analysis showed that level of p62 mRNA was not significantly down regulated by SG511-BECN (Fig. 2H). We next monitored the autophagic flux by assessing the ratio between LC3-II of p62 levels. Treatment with BafA1, an autophagy inhibitor that blocks autolysosome formation, enhanced SG511-BECN-induced LC3-II accumulation, while the SG511-BECN-induced reduction of p62 was partially prevented by BafA1 (Fig. 2I), confirming the autophagy-mediated p62 degradation. Together, these findings led us to conclude that SG511-BECN could effectively induce autophagic cell death in leukemic cells.

### Superior activity of SG511-BECN against leukemia cells *in vitro*

**Figure 3:**
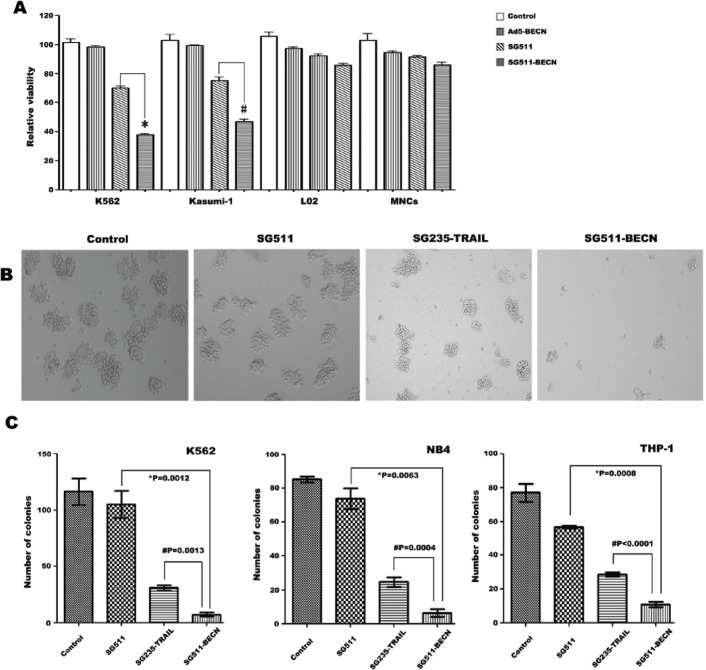
Superior efficacy of SG511-BECN compared with the CRAd expressing TRAIL (A) Leukemic cells (2×10^4^), human MNCs, and L02 cells were treated with Ad5-BECN, SG511, or SG511-BECN at an MOI of 50 for 72 h before analysis of cell viability (MTT assay). The results represent means ± SD of three independent experiments. *P<0.0001, *vs* SG511, # *P*=0.0007, *vs* SG511. (B) K562 cells were treated with the indicated viruses at 50 MOI and colonies were observed on day 7 under a light microscope. (C) K562, NB4, and THP-1 cells were infected with or without SG511, SG235-TRAIL, and SG511-BECN at an MOI of 50, respectively. The cells were then plated in methylcellulose medium. After incubation for 7 days, colonies (more than 50 cells) were scored. Data represent means ± SD for separate experiments. #SG511-BECN *vs* SG235-TRAIL, *SG511-BECN *vs* SG511.

To investigate the increased cytotoxic effect of SG511-BECN compared with SG511 and Ad-BECN, leukemia cells and human normal cells were infected with different vectors. At 72 h after viral infection, cell viability was determined by the MTT assay. Results showed that SG511-BECN had a remarkable cell killing effect compared with the other viruses in each leukemia cell line. The cell killing effect of SG511-BECN was about 2 times higher than that of SG511. In contrast, minimal cytotoxic effect was detected in normal cells (Fig. 3A). Importantly, CFU-L formation was almost completely eliminated when the SG511-BECN was used at an MOI of 50 (Fig. 3B). However, SG511 did not show a significant inhibitory effect on colony growth of K562 cells. Therefore, these findings suggest that the antileukemia activity of SG511-BECN is superior to that of SG511 vector *in vitro*.

Our previous data showed that SG235-TRAIL has an enhanced antileukemic therapeutic effect by induction of apoptosis [8]. In the present study, we compared the antileukemic activity of SG511-BECN with that of SG235-TRAIL. K562, NB4, and THP-1 cells were infected with the indicated viruses at an MOI of 50, and then colony assays were performed (Fig. 3C). Treatment with SG511 slightly inhibited colony formation of these cells; by contrast, fewer colonies formed after treatment with SG235-TRAIL, and there was an additional marked decrease in CFU-L formation upon treatment with SG511-BECN. To further assess whether enhanced antitumor activity of SG511-BECN is specific for leukemic cells, cytotoxicity of different viruses against human solid tumor cells (Hep3B, Hela, and T42) was determined by the violet assay. The results showed that cell killing by SG511-BECN was more effective than by SG511 (Supplementary Fig. 1B), suggesting the activity of SG511-BECN against a broad spectrum of human cancers.

### SG511-BECN effectively suppresses colony formation of primary CML cells from patients with imatinib resistant disease and AML cells from relapsed disease

**Figure 4:**
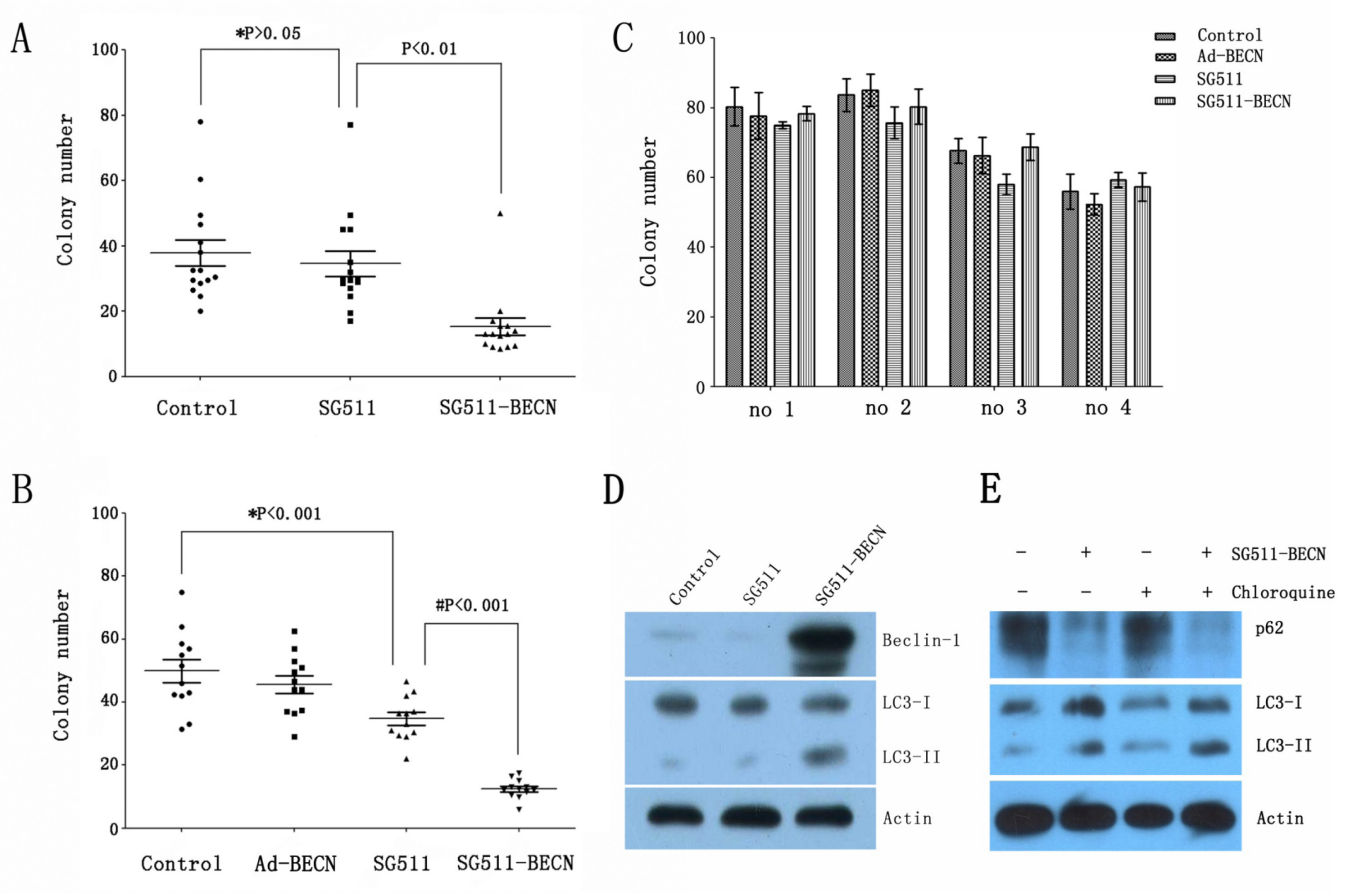
SG511-BECN induces autophagy in primary leukemic cells to preferentially inhibit CFU-L formation (A) Freshly isolated leukemia cells from CML patients (n=15) were cultured in methylcellulose medium in the presence of SG511, or SG511-BECN at an MOI of 50. After 12 days, colonies were scored. *, *P*>0.05 *vs*. Control; # *P*<0.01 *vs*. SG511. (B) Leukemic cells from 12 patients with AML were treated with the indicated vectors at an MOI of 50 before analysis of effects on colony formation. *, *P*<0.001 *vs*. Control; # *P*<0.001 *vs*. SG511. (C) Bone marrow cells obtained from 4 healthy volunteers were treated with the indicated viruses, and colonies were counted after 12 days. For all panels, values represent the mean of experiments in triplicate. (D) The primary leukemic cells from a CML patient in blast crisis (case 1) were infected with the indicated viruses at an MOI of 50, cell lysate was immunoblotted with anti-Beclin-1, anti-LC3, and anti-â-actin antibodies at day 2 of the experiment. (E) The primary blasts were treated with SG511-BECN (50 MOI), or SG511-BECN plus chloroquine (25 ìM) for 48 h. Autophagic flux was determined using Western blotting.

To determine whether SG511-BECN virus is effective against primary leukemia cells, we tested the clonogenic capacity of primary blasts isolated from patients with CML in chronic phase or myeloid blast crisis with imatinib-resistant disease, and AML patients in newly diagnosed or relasped disease. The characteristics of these cases are shown in Table 1. Primary cells were exposed to the different viruses at an MOI of 50 and subjected to blast colony assays. Results showed that exposure of primary cells to SG511 virus had relatively little effect on clonogenic potential. However, in both AML (Fig. 4A) and CML (Fig. 4B), SG511-BECN resulted in a pronounced reduction in colony formation compared to SG511 (*P*<0.01, and *P*<0.001, respectively). In contrast, the ability of normal specimens (n=4) to form colonies was not substantially affected by treatment with SG511 or SG511-BECN, respectively (Fig. 4C). Further, a dramatic increase in the Beclin-1 expression and conversion of LC3-I to LC3-II was observed in primary cells treated with SG511-BECN (Fig. 4D). Using chloroquine, an alkalinizing lysosomotropic drug, autophagic flux was determined in primary leukemic cells obtained from a CML patient. Results showed that SG511-BECN-induced LC3-II accumulation was apparently augmented in cells exposed to chloroquine (Fig. 4E), suggesting efficient autophagic flux. Collectively, these data suggest that SG511-BECN significantly impairs leukemic but not normal hematopoietic progenitor-cell function.

**Tabel 1:** Clinical characteristics of patients

patient	Sex/age	Diagnosis	Blast, %	Cytogenetics	Responses at time of sampling	Responses after the time of sampling
1	M/51	CML-BP	75	46XY,t(9;22)(q34;q11)	prior treatment: Imatinib	NA
2	F/65	CML-BP	82.3	46XY,t(9;22)(q34;q11)	prior treatment: Imatinib	NA
3	M/42	CML-BP	29	46XY,t(9;22)(q34;q11)	prior treatment: Hydroxyurea	NA
4	M/44	CML-CP	<5	46XY,t(9;22)(q34;q11)	newly diagnosed	NA
5	F/18	CML-CP	<5	46XY,t(9;22)(q34;q11)	newly diagnosed	NA
6	M/68	CML-CP	<5	46XY,t(9;22)(q34;q11)	newly diagnosed	NA
7	M/56	CML-CP	<5	46XY,t(9;22)(q34;q11)	newly diagnosed	NA
8	M/61	CML-CP	<5	46XY,t(9;22)(q34;q11)	newly diagnosed	NA
9	M/55	CML-CP	<5	46XY,t(9;22)(q34;q11)	newly diagnosed	NA
10	F/71	CML-CP	<5	46XY,t(9;22)(q34;q11)	newly diagnosed	NA
11	F/50	CML-CP	<5	46XY,t(9;22)(q34;q11)	newly diagnosed	NA
12	F/57	CML-CP	<5	46XY,t(9;22)(q34;q11)	newly diagnosed	NA
13	M/51	CML-CP	<5	46XY,t(9;22)(q34;q11)	newly diagnosed	NA
14	M/35	CML-CP	<5	46XY,t(9;22)(q34;q11)	newly diagnosed	NA
15	F/53	CML-CP	<5	46XY,t(9;22)(q34;q11)	newly diagnosed	NA
16	M/27	AML-M2	63	45X,-Y,t(8;21)(q22;q22)	newly diagnosed	no CR
17	M/66	AML-M5	46	46XY	newly diagnosed	no CR
18	F/48	AML-M5	59	46XY	newly diagnosed	CR
19	M/20	AML-M2	54	46XY	newly diagnosed	CR
20	F/24	AML-M4	50.5	46XX,t(11;12)(p15;q14)	newly diagnosed	no CR
21	M/24	AML-M3	83	46XY,t(15;17)(q22;q21)	newly diagnosed	CR
22	M/19	AML-M0	79	44/45XY,complex karyotype	relapsed	CR
23	F/21	AML-M3	96	46XY,t(15;17)(q22;q21)	newly diagnosed	CR
24	M/62	AML-M2	88.5	46XY	newly diagnosed	no CR
25	F/44	AML-M2	27	46XX	relapsed	no CR
26	F/42	AML-M2	82	46XX	relapsed	no CR
27	M/55	AML-M3	90	46XY,t(15;17)(q22;q21)	newly diagnosed	CR

F:female; M:male; BP:blast phase; CP:chronic phase; CR: complete remission; NA:not available

### Interaction of Beclin-1 with UVRAG is essential for the induction of autophagic cell death by SG511-BECN

**Figure 5:**
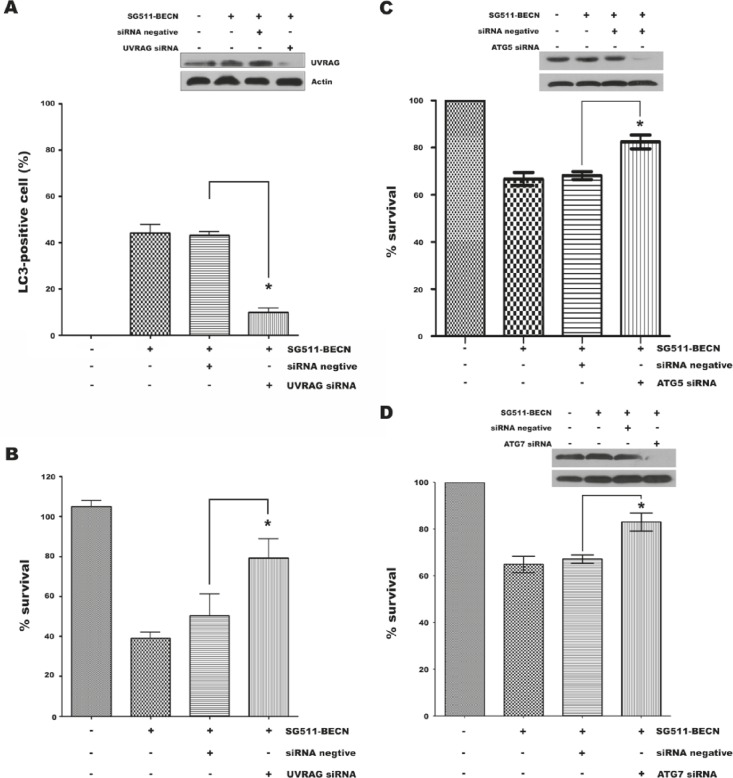
siRNA-mediated UVRAG, ATG5, or ATG7 knockdown reverses suppressive effects of SG511-BECN (A) Expression of autophagy gene UVRAG was silenced by siRNA in K562 cells containing GFP-LC3, and LC3-positive vesicle-containing cells were determined after treatment with SG511-BECN for 48 h. UVRAG expression was detected by Western blotting of K562 cells. â-actin was used as protein loading control (insert). (B) Experimental conditions as in (A). Cell viability was assessed by an MTT assay. *Represents *P*<0.001 compared to control. Standard error was calculated from three independent experiments. (C) K562 cells were transfected with 100 nM ATG5 siRNA for 48 h, and then treated with SG511-BECN for 48 h. The indicated protein level was analyzed by Western blot (insert). In parallel, cell viability was assessed by an MTT assay (*P*=0.0019). (D) ATG7 protein level and viability of K562 cells were determined after transfection with ATG7 siRNA as indicated in (C). *represents *P*<0.0029.

As UVRAG-mediated activation of the Beclin-1-PI(3)KC3 complex promotes autophagy and suppresses the proliferation of cancer cells [29], we examined the effect of siRNA against UVRAG on autophagic cell death induced by SG511-BECN to demonstrate indirectly whether overexpression of Beclin-1 contributes to cell death of leukemia. siRNA was used to reduce endogenous UVRAG expression in K562 cells infected with SG511-BECN, which resulted in a remarkable reduction of UVRAG protein level (Fig. 5A). A significantly decreased number of LC3-positive cells was observed upon siRNA treatment (*P*<0.001; Fig. 5A). Moreover, knockdown of UVRAG also resulted in reversal of the suppressive effects of SG511-BECN (*P*<0.05) (Fig. 5B). Together, these data suggest that autophagy induced by overexpression of Beclin-1 is an important mechanism by which SG511-BECN generates antileukemic activity, and the interaction of Beclin-1 with UVRAG is essential, at least in part, for the induction of autophagic cell death. Next, we targeted key components of the autophagic machinery using specific siRNAs and evaluated their effects on SG511-BECN-induced cell death. Selective knockdown of either ATG5 (Fig. 5C) or ATG7 (Fig. 5D) resulted in reversal of the suppressive effects of SG511-BECN on cell viability.

### Enhanced Anti-leukemia effect of SG511-BECN *in vivo*

**Figure 6:**
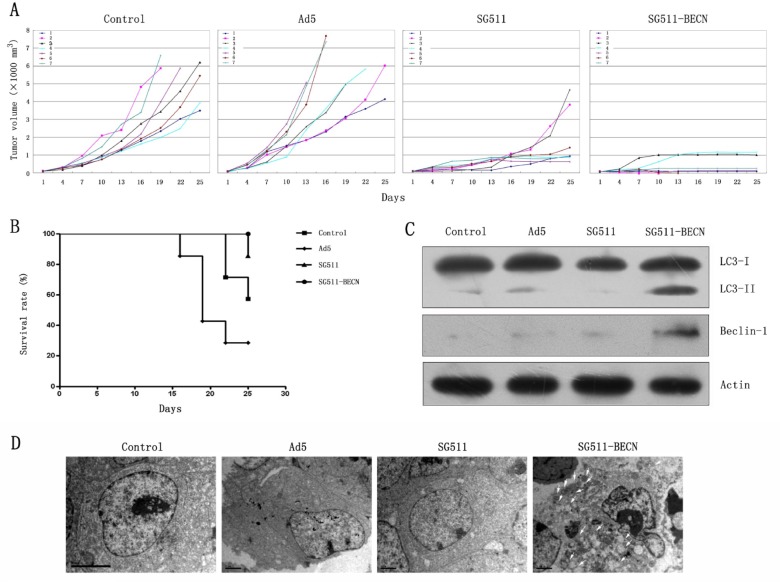
Antileukemic efficacy of SG511-BECN in established K562 tumors *in vivo* Mice bearing subcutaneous K562 xenografts (n=8) were randomized to receive one of the following intratumoral injections on 5 consecutive days: PBS, Ad5, SG511, and SG511-BECN. (A) Time courses of changes in tumor volume in each group (n=7) are shown. (B) Survival curve analysis. The percentage of surviving mice was determined by monitoring the death of mice over a period of 25 days. Mice treated with SG511-BECN shows significant survival advantage over mice treated with other groups (*P*=0.0072). (C) Tumor tissues were obtained on day 5 after treatment with different viruses, and Beclin-1 expression and the level of LC3-II was determined by Western blotting. The expression of â-actin was used as loading control. (D) Electron microscopy images were taken of tumor tissues (3700×). Autophagic vacuoles are denoted by arrow.

To assess the therapeutic efficacy of SG511 versus SG511-BECN against leukemic cells *in vivo*, K562 xenograft model was established. Figure 6A shows that CRAd-treated tumors were substantially delayed in their growth. Ad5 virus did not show any antileukemia activity. At 22 days post treatment, xenografts of mice treated with SG511 reached an average tumor volume of 1256 mm^3^. In contrast, mean tumor volume was 388 mm^3^ in the mice treated with SG511-BECN. Importantly, complete regression of the tumors was observed in two of seven mice treated with SG511-BECN, but not in SG511-treated mice. The Kaplan-Meier survival curve shows that all animals treated with SG511-BECN were still viable, while 85.7% of the SG511-treated mice survived. In comparison, only 57.1% of control mice and 28.6% of Ad5-treated mice were alive in the same time period (Fig. 6B), suggesting that CRAd expressing Beclin-1 confers significant survival benefits (*P*=0.0072). To verify induction of autophagy *in vivo*, the overexpression of Beclin-1 and LC3-I to LC3-II conversion in tumors were detected by Western blot on day 5 after viral injection. Notable overexpression of Beclin-1 protein and conversion of LC3-I to LC3-II were found in the SG511-BECN-treated tumor (Fig. 6C), confirming the role of autophagy in inhibiting tumor growth. Furthermore, TEM analyses of tumors showing the appearance of autophagic vesicles in SG511-BECN-treated group also supported this conclusion (Fig. 6D).

## DISCUSSION

Although gene-virotherapy by arming CRAds with pro-apoptotic genes is a well-established modality for various cancers, appearance of cancer cells resistant to apoptosis is one of the major concerns. Accumulating evidence indicates that autophagic cell death plays an important role in the generation of anticancer activity in apoptosis defective cells [23-25]. Autophagy also has been implicated as the main mechanism by which some antileukemia agents produce their antileukemic activities [30-33]. For example, arsenic trioxide was showed to induce autophagic cell death in leukemic cell lines and AML progenitors, which could be reversed by knockdown of Beclin-1 or Atg7 [31]. Interestingly, induction of autophagy not only mediates cell death induced by dexamethasone in lymphoid leukemia, but also is required for leukemic cells to overcome glucocorticoid resistance [30,33]. These studies raise the possibility that genes that induce autophagy could act as sensitizers of leukemia cells to the killing effects of oncolytic virus. The present study shows that SG511-BECN, a chimeric CRAd armed with Beclin-1, effectively kills a variety of leukemic cells through viral oncolysis, while production of Beclin-1 by the infected cell significantly augments the therapeutic effects. We have validated induction of autophagy by our Beclin-1-armed viruses, as indicated by the formation of the autophagic vesicles detected by four independent approaches: TEM, AVO staining, LC3-I to LC3-II conversion by Western blotting and LC3 translocation to vacuoles in GFP-LC3-transfected K562 cells, respectively. Also, the autophagic flux assays revealed that SG511-BECN induced autophagy. Consistent with these results, we found that ATG5 and ATG7 knockdown rescued the cells from SG511-BECN-induced growth inhibition. Because UVRAG, a Beclin-1-interacting protein, associates with the Beclin-1-Bcl-2-PI(3)KC3 multiprotein complex, where UVRAG and Beclin-1 interdependently induce autophagy [29], reversal of the antileukemic effects of SG511-BECN by knockdown of UVRAG also demonstrated that autophagic cell death contribute to the enhanced cytotoxicity of the virus against leukemia cells.

Human Beclin-1 is the functional homologue of yeast Vps30/Apg6 gene that interacts with class Ⅲ PI3K complex and participates in autophagic vesicle nucleation [34-36]. Beclin-1 gene knockout in mice was demonstrated to cause a marked increase in epithelial and hematopoietic malignancies [37,38]. The deletion and reduced expression of Beclin-1 was found in various types of cancer cells [39]. In patients with hepatocellular carcinoma and gastric cancers, reduced Beclin-1 expression is associated with a poor prognosis [40,41], while high expression is associated with favourable prognosis for salivary carcinoma and lymphoma patients [42,43]. Overall, the data suggest that Beclin-1 is a tumour suppressor gene. An earlier study showed that transfection of breast cancer cells that lack detectable endogenous Beclin-1 protein with Beclin-1 gene inhibited cell growth and tumor clonigenicity *in vitro* and *in vivo* [34]. As previous studies demonstrated that gene-virotherapy resulted in an augment expression of transgenes due to replication of the virus within cancer cells [16,17], the important question arises: whether integrating Beclin-1 gene therapy into an oncolytic virus elicit strong antileukemia activity? We revealed that a chimeric CRAd plus Beclin-1 achieved superior antileukemic effects and survival compared with group treated with SG511 virus alone. Notably, SG511-BECN also effectively kills leukemic progenitors evidenced by almost complete inhibition of CFU-L formation. These results support the observation that Ras-induced expression of Noxa and Beclin-1 promotes autophagic cell death and reduces clonogenic survival [44]. Furthermore, treatment with SG511-BECN induced complete elimination of established tumor xenografts in a mouse leukemia model. Together, these results suggest that CRAds armed with therapeutic transgenes such as Beclin-1 could eradicate leukemia stem cells although the exact effects of SG511-BECN on CD34^+^ leukemic stem cells has not been determined.

The relationship between autophagy and cancer, however, is still unclear [22,39,45]. Recently, numerous studies shed light onto different aspects of this relationship. Firstly, autophagy activation promotes survival of cancer cells under stress, including cytotoxic agents and different signaling pathway inhibitors [20,25,46,47]. This protective autophagic process has also been observed in AML cells [46,48]. For example, erlotinib, an epidermal growth factor receptor inhibitor, can induce autophagy in various AML cell lines by inhibiting the phosphorylation mTOR targets [48]. In several studies, hyperactivation of basal autophagy was shown to be important for the therapeutic resistance of malignancy. In support of this hypothesis, a study showed that ATG12 was upregulated in breast cancer cells and knockdown of the ATG12 gene fully suppressed the refractoriness of the cancer cells to treatment with molecularly targeted agents [49]. Recently, accumulating evidence indicates that the function of autophagy in tumor is dynamic with both protumorigenic and tumor suppressive roles which depend on stage of cancer progression, cellular context and tissue of origin [45]. In fact, induction of autophagic cell death has been demonstrated in several types of tumors with defective apoptosis. Akar *et al*. [50] revealed that siRNA of Bcl-2 in MCF-7 breast cancer cells deficient for caspase 3 inhibited cell growth and colony formation, and resulted in cell death by autophagy but not apoptosis. For leukemia, *in vivo* experiments showed that mammalian target of rapamycin inhibitor RAD001 increased survival of leukemic engrafted animals *via* induction of autophagy, but not apoptosis [51]. We and others have previous shown that CRAd-mediated TRAIL gene therapy for leukemia and breast cancer resulted in significant increases of cell killing and suppression of tumor growth by inducing apoptosis [8,52]. An interesting finding of this work is that SG511-BECN produced antitumor effects in a CFU-L formation assay that were superior to that observed by using SG235-TRAIL. Notably, we showed that efficacy of SG511-BECN was not affected by patient characteristics, such as chromosome aberrations, and imatinib-, or chemotherapy-resistance making it potentially useful in refractory or relapse leukemia patients too. Crucially, SG511-BECN treatment failed to inhibit not only proliferation of human normal MNCs and hepatic L02 cells but also normal hematopoietic colony formation, consistent with previous reports in which oncolytic adenovirus has the capability to selectively replicate in tumor cells not in normal cells in a p53-dependent [53] or -independent manner [54], suggesting that the combination selectively kills leukemia cells while sparing normal cells. Taken together, our results thus provide strong evidence that Beclin-1 gene-virotherapy is an alternative promising antileukemia therapeutic strategy.

Intravascular delivery of CRAds is required for effectively treating leukemia. Previous studies suggested that Ad-based virotherapy or genetherapy is associated with several limitations during systemic administration. These limitations include the immune attack against adenoviruses by complement, cytokine or most critically neutralizing antibodies, which is account for the virus toxicity and limited efficacy, and the sequestration of the virus by liver cells [55,56]. However, it was suggested that genetic modifications made to CRAds using tumor-specific promoters including human telomerase reverse transcriptase (hTERT) and fiber modifications might allow the escape of adenovirus from preexisting antibodies, and the selective infection of the viruses for tumor cells, but not of normal cells [55,57,58]. In the present study, the effects of SG511-BECN on human normal liver cell line L02 were investigated, no significant toxicity in L02 cells was observed, suggesting that this chimeric oncolytic virus carrying hTERT promoter might reduce hepatotoxicity. However, additional work will be needed to assess the therapeutic potential and immunogenicity of SG511-BECN during intravascular therapy.

In conclusion, the data presented herein show for the first time that, by introducing the Beclin-1 gene into the oncolytic adenoviral backbone, antileukemia activity of the virus can be significantly improved, leading to a complete elimination of leukemia xenografts and prolonged survival of tumor-bearing mice. Thus, CRAd with Ad5/11 fibers, expressing the Beclin-1 gene may offer a novel promising gene-virotherapy for the treatment of leukemia via a unique mechanism of action distinct from apoptosis.

## MATERIALS AND METHODS

### Normal and leukemia patient samples, cell lines, and reagents

Bone marrow samples were obtained from healthy controls and patients with chronic myeloid leukemia (CML; n=15) and acute myeloid leukemia (AML; n=12). The mononuclear cells (MNCs) were isolated by Ficoll-Hypaque density gradient centrifugation after informed consent was obtained using guidelines approved by the Ethics Committee of Zhejiang University. Human AML cell lines NB4, THP-1 and CML cell line K562 were purchased from the American Type Culture Collection (ATCC; Manassas, VA, USA), and AML cell lines Kasumi-1 and KG-1 was kindly provided by Prof. S Chen (Shanghai Jiaotong University, Shanghai, China) and Prof. R Xu (Zhejiang University, Hangzhou, China), respectively. L-02 cell line, a normal human liver cell line, was purchased from the Shanghai Cell Collection (Shanghai, China). The cell lines were authenticated by comparing immunophenotype, karyotype, and molecular marker. All the cell lines were used within 6 months after documentation, and cultured as described earlier [8]. The pancaspase inhibitor z-VAD-fmk was purchased from R & D (Minneapolis, MN, USA), Bafilomycin A1 (BafA1) and chloroquine were purchased from Sigma (St Louis, MO, USA).

### Construction of recombinant viruses

The complete cDNA sequence of Beclin-1 gene was amplified by PCR by using the upstream primer (5'-CCG GAA TTC ACC ATG GAA GGG TCT AAG ACG TCC AAC-3') and downstream primer (5'-ACG CGT CGA CTT ATC ATT TGT TAT AAA ATT GTG AGG-3'). The synthetic DNA was released with *EcoR I* and *Sal I* (New England Biolabs, Beverley, MA, USA) and ligated into plasmid pENTR-12 to generate pENTR12-BECN. After sequence confirmation, pENTR12-BECN construct was then recombined using the LR reaction (Invitrogen, Carlsbad, CA, USA) into the pPE11 to form pPE11-BECN. pPE11 is an adenovirus packaging plasmid construced by replacing Ad5 fiber with Ad5/11 chimeric fiber [3]. Finally, the pPE11-BECN was transfected into HEK293 cells using Lipofectamine 2000 (Invitrogen) together with plasmid pSG500 [2]. The obtained CRAds was named as SG511-BECN. Enhanced green fluorescence protein (GFP) gene was cloned into SG511 to construct SG511-GFP and SG235-TRAIL was constructed as described previously [8]. Ad-BECN, a replication-deficient adenovirus expressing Beclin-1, was provided by Prof. Q Qian (Second Military Medical University, Shanghai, China).

### Cell proliferation assay

For cell proliferation assays using 3-(4,5-dimethylthiazol-2-yl) -2,5-diphenyltetrazolium bromide (MTT; Sigma), cells were seeded in 96-well plates at a density of 2×10^4^/ml in the presence or absence of Ad-BECN, SG511, or SG511-BECN at a concentration of 50 MOI. After 48 h, 20 ìl of MTT solution (5mg/ml) was added to each well. The samples were incubated at 37℃ for 4 h and the absorbance was measured at 570 nm by spectrophotometry.

### Flow Cytometric Analysis

After treatment with SG511-BECN for 48 h, cells were harvested and washed with PBS buffer containing 5 mmol/L EDTA. And then incubated at 37℃ for 30 min. Cell death was determined by staining cells with annexin V-FITC and propidium iodide (PI) using annexin V-FITC apoptosis detection kit (BD Pharmingen, San Diego, CA, USA) followed by analysis on a BD FACSCalibur flow cytometer. Expression of the GFP gene was also assessed by the FACSCalibur.

### Detection of autophagosome formation with acridine orange

To detect the presence of acidic vesicular organelles (AVOs), leukemic cells were treated with SG511, and SG511-BECN, respectively. After 48 h, the cells were stained with the vital dye acridine orange (1 ìg/mL; Molecular Probes, Eugene, OR, USA) and then examined immediately by fluorescence microscope (IX71; Olympus, Tokyo, Japan) with a red filter (excitation 560 nm, emission 645 nm).

### Staining autophagosomes with GFP-LC3

K562 cells stably expressed GFP-LC3 were established as described previously [20]. The cells were treated with SG511 or SG511-BECN at an MOI of 50 for 48 h. The fluorescence of GFP-LC3 was viewed under a fluorescent microscope and photographs were taken. The cells with GFP-LC3 vacuoles (autophagosomes) were counted by image analysis using ImageJ software (National Institutes of Health, Bethesda, MD, USA).

### Cellular localization studies

K562 cells were infected with or without SG511-BECN at MOI of 50 for 48 h and cells were washed with PBS, fixed in the 0.1% poly-L-lysine-treated slides with 4% paraformaldehyde, permeabilized by 0.1% Triton X-100 and then incubated with anti-Beclin-1 (Novus Biologicals, Littleton, CO, USA), and calreticulin (Merck, Calbiochem, Darmstadt, Germany) at 4℃ for overnight. After washing with PBS, the slides were incubated with Alexa Fluor 488 and Alexa Fluor 568 (Invitrogen), and examined for visualization under an Olympus confocal microscope (FV1000).

### Transfection of UVRAG siRNA vector

K562 cells expressing GFP-LC3 were transiently transfected with human UVRAG-siRNA, ATG5 siRNA, or ATG7 siRNA, respectively, as per the manufacturer's instructions, using shRNA Plasmid Transfection Reagent (Santa Cruz Biotechnology, Santa Cruz, CA, USA) or Lipofectamine Transfection Reagent (Invitrogen). Control-siRNA (Santa Cruz) was used as negative control. After transfection, cells were cultured for 48 h, and treated with SG511-BECN, and the viability of cells, protein expression of the targeted genes, and GFP-LC3 vacuoles were analyzed.

### Western blot Analyses

Western blot analyses were performed as described previously [8,20]. The primary antibodies used in this study were purchased from: anti-Beclin-1 (Novus Biologicals), anti-LC3 (Novus Biologicals), anti-p62, anti-caspase-3, -9, and -8, anti-PARP, anti-ATG5, and anti-ATG7 (Cell signaling, Beberly, MA, USA), anti-E1A (BD PharMingen), anti-UVRAG (Sigma), and anti-â-actin (Santa Cruz).

### Real-time RT-PCR

Total RNA was isolated and quantitative real-time PCR (qRT-PCR) was performed as previously described [25] using the primers: 5'-TGG TAG GAA CCC GCT ACA AGT-3' (forward) and 5'-CCC GAA GTG TCC GTG TTT C-3' (reverse) for human p62 and 5'-ATG GGG AAG GTG AAG GTC G-3' (forward) and 5'-GGG TCA TTG ATG GCA ACA ATA TC-3' (reverse) for GAPDH.

### Colony forming analysis

Leukemic cell lines, MNCs from the patients with AML, CML or healthy controls were infected with or without the indicated viruses (50 MOI) and seeded in triplicate in a mixture containing 1.35% methylcellulose (Sigma) in Iscove's modified Dulbecco's medium (IMDM; Sigma) supplemented with 20% fetal bovine serum (Hyclone, Logan UT, USA) and10^−4^ M 2-Mercaptoethanol (Sigma). For colony assay of bone marrow cells, 3 U/mL recombinant human (rh) erythropoietin, 50 ng/mL rh stem cell factor, 30 ng/mL rh granulocyte macrophage–colony-stimulating factor, and 10 ng/mL rh interleukin-3 (Peprotech, Rocky Hill, NJ, USA) were added to the methylcellulose medium. The colonies were evaluated under a microscope on day 12 of culture. In contrast, leukemic cell lines were seeded in cytokine-free mixture as described previously [26], and the colonies were counted on day 7.

### Transmission electron microscope

Tumors or leukemic cells were harvested after treatment with the viruses, and fixed with 2.5% phosphate-buffered gluteraldehyde, and then postfixed in 1% phosphatebuffered osmium tetroxide, embedded in Spurr's resin. The ultrathin sections (0.12 ìm) were cut, double stained with uranyl acetate and lead citrate. Representative areas were chosen and viewed with a Philips TECNA10 transmission electron microscope (TEM) at accelerating voltage of 80kV.

### Animal experiments

All animal experiments were approved by the Institutional Animal Care and Use Committee, Zhejiang University. Female severe combined immunodeficient mice (3- to 4-weeks-old) were purchased from Experimental Animal Center of the Chinese Academy of Sciences (Shanghai, China). K562 cells (1×10^7^) were injected subcutaneously into the lower right flank of mice. When the s.c. nodules grew and reached a volume of 80-100 mm^3^, the mice were randomly assigned to three treatment groups (Ad, SG511, and SG511-BECN) and one control group (n=8). A daily dose of 2×10^8^ plaque-forming units of different adenoviruses in 100 ìl of PBS, or PBS alone, was administrated intratumorally for 5 days. Tumor growth was monitored and measured every 3 days with a Vernier caliper and tumor volume was calculated as previously described [8]. On day 5 after treatments, one mouse from each group was randomly selected, and humanely killed. Tumors were harvested for Western blotting and TEM analysis.

### Statistics

The significance of differences between groups was assessed by Student's unpaired two-tailed *t* test. The antileukemia effect *in vivo* was evaluated by plotting survival curves according to the Kaplan-Meier method, and survivals among treatments groups were compared using the log-rank test. A *P*-value of <0.05 was considered significant.

## SUPPLEMENTAL INFORMATION


