# Supplementary material for: Potent antitumor activity of oncolytic adenovirus expressing Beclin-1 via induction of autophagic cell death in leukemia
Source: Oncotarget. 2013 Jun 3;4(6):860–74. doi: 10.18632/oncotarget.1018 (PMC3757243; doi:10.18632/oncotarget.1018)
Supplement: Supplementary file 1 [file oncotarget-04-860-s001.docx]

**
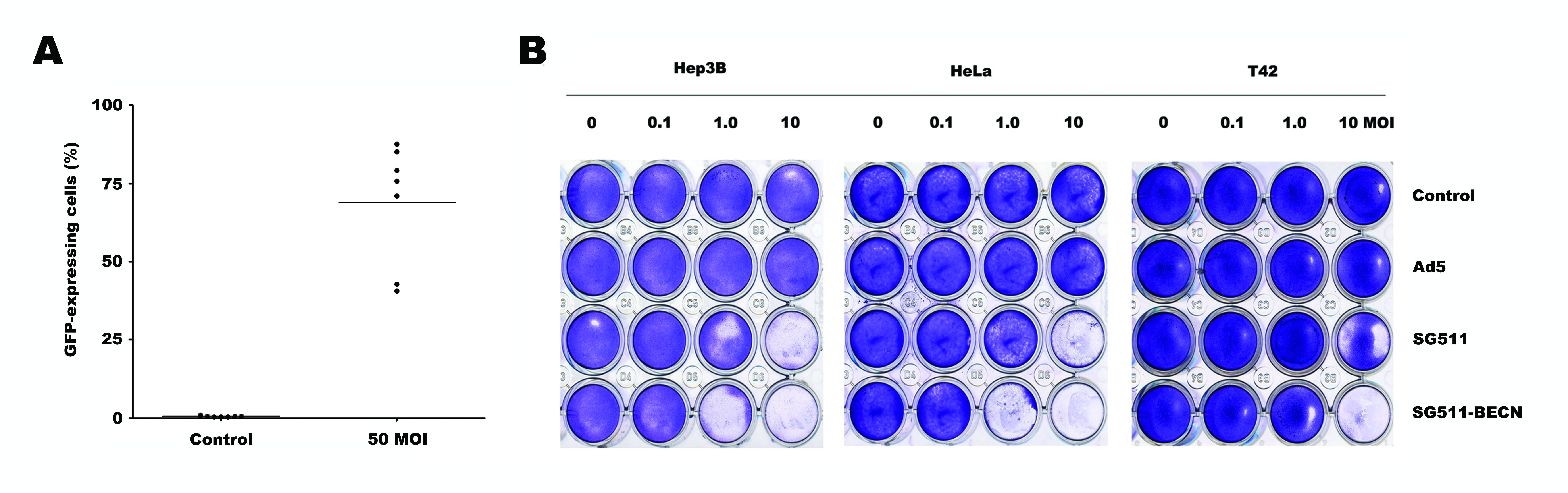
Supplementary Figure 1.** (**A**) Infection of SG511 virus in primary leukemic cells. Freshly isolated leukemic cells obtained from 7 patients with AML were infected with or without SG511-GFP at an MOI of 50 for 48 h, and subjected to flow cytometric analysis. (B) Human Hep3B, HeLa, and T42 cell lines were treated with increasing concentrations of the indicated viruses for 72 h, and then stained with 2% crystal violet. After washing with water, air-dried dishes were photographed by a digital camera.
